# Coping with COVID: How a Research Team Learned To Stay Engaged in This Time of Physical Distancing

**DOI:** 10.1128/mBio.00850-20

**Published:** 2020-04-17

**Authors:** Danica M. Sutherland, Gwen M. Taylor, Terence S. Dermody

**Affiliations:** a Department of Pediatrics, University of Pittsburgh School of Medicine, Pittsburgh, Pennsylvania, USA; b Center for Microbial Pathogenesis, UPMC Children’s Hospital of Pittsburgh, Pittsburgh, Pennsylvania, USA; c Department of Microbiology and Molecular Genetics, University of Pittsburgh School of Medicine, Pittsburgh, Pennsylvania, USA

**Keywords:** COVID-19, pandemic, mentoring, physical distancing, research lab, SARS CoV-2, social distancing, virology

## Abstract

Physical distancing imposed by the COVID-19 pandemic has led to alterations in routines and new responsibilities for much of the research community. We provide some tips for how research teams can cope with physical distancing, some of which require a change in how we define productivity. Importantly, we need to maintain and strengthen social connections in this time when we can’t be physically together.

## EDITORIAL

We study reovirus and chikungunya virus in our laboratory with an overall goal to better understand the replication and pathogenesis of these viruses. As important as it is, our work is not essential in the setting of the COVID-19 pandemic. Therefore, like thousands of laboratories around the world, the COVID-19-imposed “physical distancing,” a term we prefer to “social distancing,” has led us to pause our research. However, physical distancing does not mean that we must stop engaging as a team. In fact, in this time of physical distancing, we need to be socially connected more than ever.

The suddenness of the physical distancing restrictions meant that experiments in progress had to be rapidly completed, new experiments could not be started, animal breeding had to be minimized, and personal and professional travel plans were curtailed. Those who just joined us could not start initial experiments; others with studies an experiment or two away from publication had to stand down. All too quickly, we lost our routines and sense of purpose. Moreover, the uncertainty about what the future might hold for us and those close to us has engendered considerable anxiety.

In an attempt to alleviate some of these concerns, our team has used this distancing time in a variety of ways to remain productive, scientifically and in other important ways, and engage with each other. We offer seven tips (with a nod to Stephen Covey) that might be useful for other research teams in this time of COVID. We have 15 people in our lab, but our ideas should scale for any size group or could be used to join different research teams together.

## TIP 1: ACCEPT AND ADJUST

We first must come to accept that the world has changed around us and that we must adapt and change with it. For some, this acceptance arrived all at once, but for others, it came slowly or in stages. Every day is different and may provide new challenges. No matter what role you hold in a team, it is important to remember that everyone will take this step differently. Ask how people are feeling, offer support, and share your experiences.

## TIP 2: DISTANCING IS DIFFICULT

Disasters caused by floods, hurricanes, tornadoes, and the like usually have a way of bringing us together, but when the disaster is caused by a contagion, it pushes us apart. We emphasize in every meeting that we are practicing physical distancing and not social distancing. We need one another’s support, and we must maintain (and even establish new) social interactions.

At the suggestion of our colleague Julie Pfeiffer, we established a Slack account—an alternative to e-mail—with channels designed for specific needs (e.g., science, writing, self-care, selfies, you name it), all with the goal to connect the lab. Members of our lab use this platform to message individuals or groups about projects or share strategies to evade the COVID blues—encouraging thoughts, funny memes, and pictures of our new “coworkers” (pets or children) as they climb all over us. A Slack page dedicated specifically to COVID-19 allows people to share news and journal articles for those who want it, while allowing others to take a break from this information.

## TIP 3: EMBRACE TECHNOLOGY

Like many, we have altered our lab’s meeting arrangements. This has not always been easy, especially for those of us who are technologically challenged, but it is better to see a colleague with a little technical difficulty than to not see her or him at all. Journal clubs, lab meetings, and one-on-one interactions are now held via Skype, Teams, or Zoom. We love the Zoom “gallery view” for larger groups, which allows us to simultaneously see everyone’s face on the call. Slides can be shared easily in Teams or Zoom for journal clubs (which we use to present articles on subjects other than COVID). While our lab meetings have changed focus and format, transitioning from research-in-progress presentations to updates about COVID, new communication platforms, and wellness strategies, the sense of community that this new meeting technology brings into our homes has been invaluable.

## TIP 4: GET WRITING

COVID-imposed physical distancing creates time for writing, reading, analysis, and planning. Most members of our team are working on at least one writing project. Whether it is a strengths, weaknesses, opportunities, and threats (SWOT) analysis for a new student picking a project, a thesis, a grant application, a research manuscript, an opinion piece, or a review article, think hard about where you can make the most impact, develop a plan, and get started. Our lab uses “writing sprints” to set accountability goals for writing and obtaining feedback on drafts. One “writer” and two to three “readers” develop a timeline for a writing project. The writer provides text in sections at regular intervals for critical input from the readers. The writer and readers meet virtually as a group, discuss the text, and set goals for the next scheduled meeting. Our team finds the routine established by the sprints especially welcome.

## TIP 5: GET CREATIVE

Since the main aspects of our job require reagents and instruments that are not currently accessible, we have been reevaluating approaches to success, which has required some creativity. For young trainees who may be farther away from an independent writing project (e.g., research assistants and new students), we are hosting several types of small-group sessions. Journal club presentations by these trainees focus on classic papers in our field or those related to an anticipated project. This approach ensures that the trainee understands these papers well and provides teaching opportunities for more senior members of the lab who ask questions and provide connections to more contemporary studies.

We also instituted weekly “Toolbox” classes hosted by senior members of the lab, in which a single technique is chosen, and online materials are provided to the junior trainees. The following week, a junior trainee presents various aspects of the technique—how to go about it, factors to consider, controls, alternative approaches, etc. The junior trainees benefit from focused learning, accountability, and presentation opportunities, while the more senior hosts gain experience teaching and training. A variation on this theme is for a senior trainee to conduct weekly sessions on a general area of interest. One of our senior graduate students is leading an informal seminar on basic principles of immunology and host defense. Members of our group who have new COVID-imposed responsibilities (like the parents who are now serving as teachers for their children) can take advantage of these opportunities by picking familiar papers and techniques.

To complement these interactions, we instituted a Virtual Coffee Shop, which is a regular, biweekly time hosted by video (people can turn off the video or mute if they wish) for accountable group writing or working. Think of it like writing in a Starbucks, but more affordable. It is important to emphasize that these activities can be customized for each lab member according to their social preferences. Some enjoy connecting using a video platform, while others find different ways to interact.

## TIP 6: EXPAND YOUR VIEW OF PRODUCTIVITY

Accept that you might not be as productive as you would like during this time. Life has changed for all of us, some substantially more than others. Alterations in our routines imposed by COVID distancing have made it hard to remain scientifically productive. However, this new reality does not equate to lack of productivity. Instead, it means that the lens of productivity must be broadened. Caring for an elder, collecting food for those in need, assembling personal protective equipment for donation, or teaching your child how to multiply complex fractions, as our colleague Carolyn Coyne succeeded in doing last week, is among your accomplishments for the day. And all that, and much more, counts as a win. Finally, when considering work, remember to set boundaries. Work and home now occur within the same four walls, which makes it easy to extend the workday well into time that should be yours, which leads us to Tip 7.

## TIP 7: TAKE CARE OF YOURSELF AND OTHERS

Most important of all, take care of yourself and take care of others. We will get through this physical distancing by working together. We reinstituted our lab’s book club and picked *Red Rising* by Pierce Brown as our first book. It’s a little dystopian, so wish us luck. One of our Slack channels is designed for exercise buffs and yogis. We started virtual happy hours, which are used by many groups, as an informal way to check in. We now dedicate time in meetings to sharing what brings us joy. We offer a few thoughts from our group. Cook an amazing meal, preferably one that takes a while to prepare. Play music, and if you don’t play, learn. Spend a little time each day exercising. Sunshine is good for the soul. Call a long-lost friend or member of your family with whom you have been out of touch. And last, express gratitude, freely and frequently.

This time of COVID offers opportunities for us all. These opportunities were not anticipated or necessarily desired. Strategies for taking advantage of these opportunities will vary for each of us, and no single path forward is perfect. But engaging as best as we can in this time of physical distancing will allow us to emerge stronger on the other side.

Be well.

